# A Single Stage and Single View 3D Point Cloud Reconstruction Network Based on DetNet

**DOI:** 10.3390/s22218235

**Published:** 2022-10-27

**Authors:** Bin Li, Shiao Zhu, Yi Lu

**Affiliations:** School of Computer Science, Northeast Electric Power University, Jilin 132011, China

**Keywords:** 3D reconstruction, single view, single stage, point cloud

## Abstract

It is a challenging problem to infer objects with reasonable shapes and appearance from a single picture. Existing research often pays more attention to the structure of the point cloud generation network, while ignoring the feature extraction of 2D images and reducing the loss in the process of feature propagation in the network. In this paper, a single-stage and single-view 3D point cloud reconstruction network, 3D-SSRecNet, is proposed. The proposed 3D-SSRecNet is a simple single-stage network composed of a 2D image feature extraction network and a point cloud prediction network. The single-stage network structure can reduce the loss of the extracted 2D image features. The 2D image feature extraction network takes DetNet as the backbone. DetNet can extract more details from 2D images. In order to generate point clouds with better shape and appearance, in the point cloud prediction network, the exponential linear unit (ELU) is used as the activation function, and the joint function of chamfer distance (CD) and Earth mover’s distance (EMD) is used as the loss function of 3DSSRecNet. In order to verify the effectiveness of 3D-SSRecNet, we conducted a series of experiments on ShapeNet and Pix3D datasets. The experimental results measured by CD and EMD have shown that 3D-SSRecNet outperforms the state-of-the-art reconstruction methods.

## 1. Introduction

In many tasks, such as virtual reality [1], experimental assistance [2], and robot navigation [3], a detailed 3D model is required, however, the facilities required to sample 3D models from the real world are costly. Moreover, it is uneconomical to manually reconstruct 3D models from 2D maps on a large scale. Many researchers proposed methods to reconstruct 3D models from a single image [4,5,6].

There are mainly two structures to represent a 3D model, voxel and point cloud. The former is just like 2D pixels but fits 3D objects into grids, sometimes containing other information such as features. It is also a regular data structure so many successful 2D methods can be easily applied. Many approaches [7,8] have focused on the voxel grid as output. However, the computational cost increases cubically to perform better geometric information or apply convolution methods.

Point cloud represents geometric information by a set of data points, each point represented by (x, y, z). Fan et al. [4] firstly applied deep learning methods to generate point clouds and proposed the chamfer distance and Earth mover’s distance, but there are many methods to improve its evaluation results. 3D-LMNet [5] used an autoencoder to design a two-stage point cloud construction network. Most of the existing works are similar to 3D-LMNet, which are multi-stage point cloud generation networks. These multi-stage networks inevitably suffered feature loss and were considered a waste of time. At the same time, the existing work often pays more attention to the structure of the point cloud generation network, while ignoring the feature extraction of 2D images.

The main tasks of point cloud reconstruction are: (1) To retain more details or small targets in the image when extracting 2D image features in order to obtain a better reconstruction effect. (2) To generate a point cloud through a simple network structure to reduce the loss of features in the process of transmission in different network stages. We propose an end-to-end point cloud reconstruction network called 3D-SSRecNet, which applies DetNet [9] as an image feature extractor and gains detailed features.

The key contributions of our work are as follows:

1. We propose a one-stage neural network for 3D reconstruction from a single image, namely, 3D-SSRecNet. 3D-SSRecNet takes an image as input and directly outputs the predicted point cloud without further processing.
2. 3D-SSRecNet includes feature extraction and 3D point cloud generation. The feature extraction network is better at extracting the detailed features of the 2D input. The point cloud generation network has a plain structure and uses a suitable activation function in its multi-layer perceptron, which reduces the loss of features during forwarding propagation to obtain an elaborate output.
3. Experiments on ShapeNet and pix3D dataset have shown that 3D-SSRecNet outperforms the state-of-art reconstruction methods for the task of single-view reconstruction. At the same time, we also proved the validity of the activation function of the point cloud generation network through experiments.

## 2. Related Work

The technology of reconstructing 3D models from 2D images has many practical applications. Therefore, 2D to 3D reconstruction technologies applicable to different application scenarios will be quite different. For example, in [2], a method is proposed to predict the liquid or solid in transparent vessels to XYZ maps. This method can be applied, for example, to the task of a robot arm taking containers and pouring liquid. Therefore, this study proposed a scale-invariant loss so that the predicted scale of XYZ map can conform to the original 2D input scale. Part-Wise AtlasNet proposed in [10] can output 3D reconstruction with a fine local structure. This is because each neural network of Part-Wise AtlasNet is only responsible for reconstructing a specific part of the 3D object. Part-Wise AtlasNet has achieved a very refined reconstruction effect, but the reconstruction process is very time consuming. Part-Wise AtlasNet is obviously more suitable for tasks such as high-precision reconstruction and the display of cultural relics. In order to obtain 3D data in real time and accurately, hardware assistance is required in addition to consideration of the camera parameters. For example, SLAM [11] constructs a 3D map by positioning the camera in real time. In virtual reality or game modeling tasks, a point cloud reconstruction network may be required to reconstruct point clouds with reasonable contour and shape without considering hardware parameters. The 3D-SSRecNet proposed in this paper is more suitable for virtual reality or game modeling scenarios. Here, we summarize the references similar to the application scenarios of 3D-SSRecNet. Due to the development of deep learning [12] and big data [13], great progress has been made in this field, and numerous valuable research has emerged. These studies can be roughly divided into 2D images to voxels and 2D images to point clouds.

From 2D images to voxels. Several approaches focus on generating voxelized output representations. V3DOR [14] applied autoencoders and variational autoencoders to generate a smoother and high-resolution 3D model. The encoder aims to learn latent representation from the image and the decoder tries to obtain corresponding 3D voxels. Xie et al. [15] applied ResNet [16] as a part of the autoencoder and proposed a multi-scale context-aware fusion module to gain better results with more views of the object. Han et al. [17] proposed a novel shape representation that enabled a tube-by-tube manner via discriminative neural networks. Based on the network, they proposed an RNN-based model to gain the 3D corresponding representation from the input image. TMVNet [18] applied the transformers to the encoder and proposed a 3D feature fusion layer to refine the predictions. Kniaz et al. [19] proposed an image-to-voxel translation model which applied a generative adversarial network. Sym3DNet [20] applied a symmetry fusion step and perceptual loss to apply symmetry prior. Yang et al. [21] designed a memory-based framework to obtain a heavily occluded 3D model to handle challenging situations. However, voxel reconstruction gains sparse space information and has a high costs. It is difficult to both predict higher resolution 3D models and process them efficiently.

From 2D images to point clouds. Fan et al. [1] designed a framework called PSGN, which firstly applied deep learning methods to the point sets generation problem. They proposed chamfer distance and Earth mover’s distance to judge the distance between point sets. 3D-LMNet [2] trained a point cloud autoencoder, then try to map images to corresponding learned embedding and they proposed diversity loss for uncertain reconstruction. 3D-ARNet [22] combined an autoencoder and a point prediction network. After the image is input into the image encoder, a simple point cloud is obtained. Pumarola et al. [23] proposed a conditional flow-based generative model to generate a map from image to point cloud, which is different from other generative models such as VAEs or GAN. Hafiz et al. [24] proposed the SE-MD network. SE-MD uses an autoencoder network as the feature extraction network and multiple decoding networks as point cloud generation networks. The final result can be obtained by fusing all the outputs of all the point cloud generation networks. However, these multi-stage models may suffer more cost of computational resources and feature loss when the feature maps propagate across networks. 3D-ReConstnet [6] applied the residual network to extract the features from the input image and used MLPs to predict point sets and, meanwhile, learned Gaussian probability distribution to refine the self-occluded part of an object and then directly applied MLPs to predict the point cloud. Ping et al. [25] projected the predicted point cloud and tried to fit edge details with ground truth. In order to enhance the features of 2D images, 3D-FEGNet [26] adds an edge extraction module to the feature extraction network. After comparing the reconstruction results of the above networks, we found that the key to a better reconstruction network are: (1) the feature extraction part of the network reflects more detailed 2D image features; (2) the loss of the extracted two-dimensional image features in the network transmission is minimized. This paper designs a single-stage point cloud reconstruction network and uses DetNet, which can retain more detailed features, as the feature extraction network.

## 3. Approach

### 3.1. Architecture of 3D-SSRecNet

**Figure 1:**
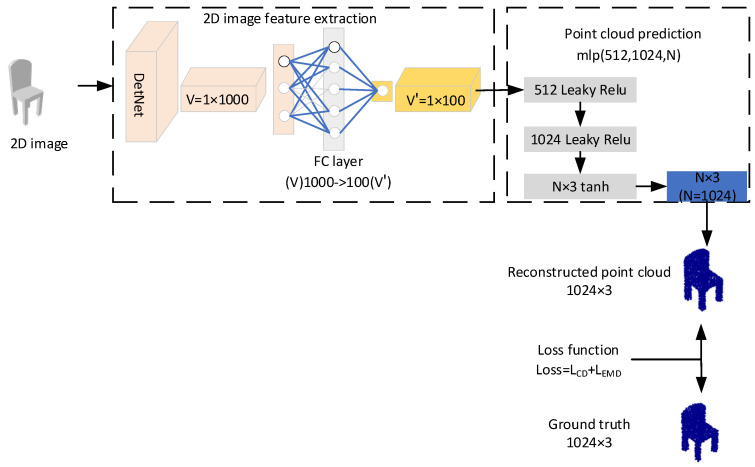
Architecture of 3D-SSRecNet.

The architecture of 3D-SSRecNet is shown in Figure 1. 3D-SSRecNet has two main parts: a 2D image feature extraction network and a point cloud prediction network. These two parts constitute a simple single-stage point cloud reconstruction network. The single-stage network structure only transfers the features of 2D pictures within the network of 3D-SSRecNet. Compared with the two-stage reconstruction network that needs to transmit features across the network, this network structure reduces the loss of features.

Given a 2D image, firstly, we obtain a latent representation V by DetNet. Then, we map V to a low dimensionality feature V’ by a full connection (FC) layer. A multi-layer perceptron (MLP) is directly applied to predict a point set afterward. During training, chamfer distance and Earth mover’s distance loss function are computed, and the update of trainable parameters is supervised.

### 3.2. 2D Image Feature Extraction

Many image feature networks applied downsampling, which brought a higher receptive field, but unavoidably caused the loss of image details. However, for reconstruction, image details are crucial for the recovery of geometric shape. This kind of network is more suitable for image classification tasks, but not for reconstruction tasks that require more detailed features.

DetNet [9] not only retains more details but also retains a large receptive field. Although DetNet was designed for object detection, its novel dilated bottleneck structure provides high-resolution feature maps and a large receptive field. We use DetNet as the backbone of image feature extraction. DetNet follows the same structure as ResNet-50 [16] until state 4, so DetNet also has the advantage of being easy to train and will not fall into gradient disappearance. Table 1 shows the parameters of the last two stages of DetNet, that is, the differences between DetNet and ResNet-50. After stage 4, DetNet keeps the size of the feature map at 16 × 16, which enables more details to be retained. The fifth and sixth stages of DetNet are composed of bottlenecks with dilated convolution, and some bottlenecks have 1 × 1 convolutions on their shortcut connections. Dilated convolution increases the receptive field. However, considering the amount of computation and memory, stage 5 and stage 6 set the same channel number of 256. At the end of the baseline, a fully connected layer is applied.

As shown in Figure 1, after feature extraction of the input image, we obtain a 1000-dimensional latent feature V of the input image. After that, the full connection (FC) layer compresses the dimension of vector V from 1000 to 100 and obtains vector V’.

### 3.3. Point Could Prediction

**Figure 2:**
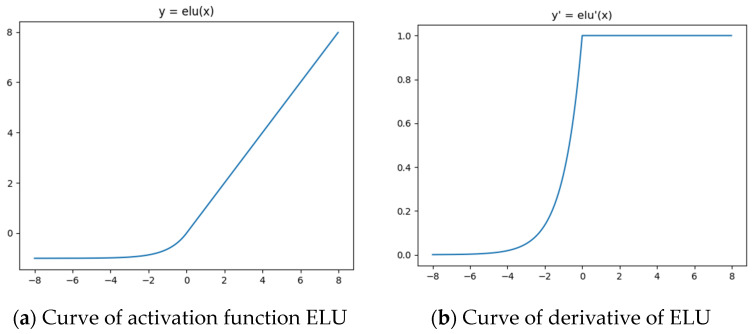
ELU activation function and its derivative.

**Figure 3:**
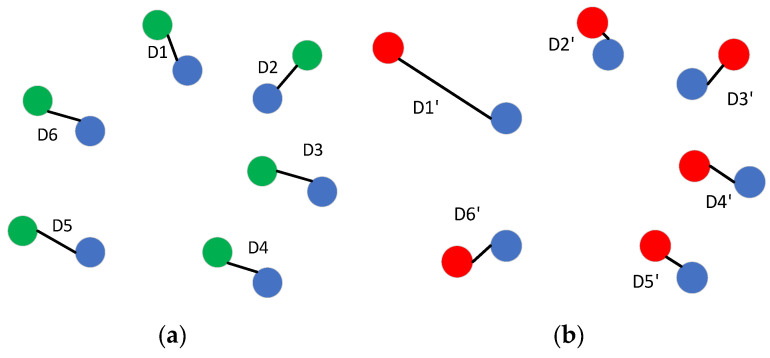
Different reconstructions with the same CD loss value.

We use three-layer MLP to predict the point sets directly. The dimensions of the outputs of the three MLP layers are 512, 1024, and N × 3, respectively. The output of the feature extraction network: vector V’ is fed into MLPs of the point cloud prediction network. On the first two layers, ELU [27] is introduced as an activation function which is defined as:

(1) $$y=\mathrm{elu}\left(x\right)=\left\{\begin{array}{c} x,\;x\geq 0\\ \alpha \left(e^{x}-1\right),x<0 \end{array}\right.$$

where parameter α is set to 1. The curves of ELU activation function and its derivative are shown in Figure 2a,b, respectively. For common activation functions, such as ReLU, the value corresponding to the negative axis is 0. However, the normalized point cloud coordinate interval is [−1, 1], which indicates that the point cloud coordinates will have negative values. As shown in Figure 2a, the value corresponding to the negative axis of the ELU activation function is non-zero. Therefore, using ELU as the activation function, the negative value information in the reconstructed network will not be lost in the forward propagation process. As shown in Figure 3b, the derivative of ELU is also non-zero on the negative axis. In the backpropagation process of the network, the negative gradient will not be lost, and it can help update the network weight.

In the experimental part, we prove that the reconstruction effect of using ELU as the activation function is better than that of using other activation functions. We directly output the predicted point sets after the last activation layer. It is actualized by the tanh function, whose outputs belong to [−1, 1], which is the same as the required point set data.

### 3.4. Loss Function

To define the loss function of reconstruction of point clouds. We have to consider two important properties. (1) Point cloud is an unordered point set so we shall obtain the same data despite how we change the order of points. (2) The geometric feature of a real object shall not change significantly regardless of any rotation transformations. However, for instance, the point coordinates seem different as we take a rotation transformation. Fan et al. [4] proposed the use of the chamfer distance (CD) and Earth mover’s distance (EMD) which satisfied the requirement. The chamfer distance is defined below.

(2) $$\;L_{CD}\left(X_{P}^{*},X_{P}\right)=\sum_{x\in X_{P}^{*}}\underset{y\in X_{P}}{min}||x-y|{|}_{2}^{2}+\sum_{y\in X_{P}^{*}}\underset{x\in X_{P}}{min}||x-y|{|}_{2}^{2}$$

It is a differentiable function with respect to point locations which are respectively calculated for each point, and it obtains the distance between it and its nearest point in the ground truth.

The Earth mover’s distance is defined below.

(3) $$L_{\mathrm{EMD}}\left(X_{P}^{*},X_{P}\right)=\underset{\varphi :X_{P}^{*}\to X_{P}}{min}\sum_{x\in X_{P}^{*}}||x-\varphi \left(x\right)|{|}_{2\;}$$

It is a measure between two distributions, so it can be considered as a “distance” between two point sets, and it constitutes the mapping between two point sets to guarantee the unique output.

Many research on point cloud reconstruction [5,17] point out the characteristics of CD and EMD: CD is related to the contour of the reconstructed point cloud. Lower CD values result in better point cloud contours. The 3D reconstruction network trained by CD can more easily capture the rough contour of objects in 2D images. However, the reconstructed network trained by CD can easily produce splash point clouds, but the visual effect is not good.

We use Figure 3 to explain the cause of point clouds. In Figure 3, the blue dot represents the ground truth, and the green dot and red dot represent two different reconstruction point clouds, respectively. D1~D6 respectively represent the distance between the six points obtained from the first reconstruction and their corresponding ground truth points. D1’~D6’ respectively represent the distance between the six points obtained from the second reconstruction and their corresponding ground truth points. We can see that the reconstruction result represented by the green point in Figure 3a is better than the reconstruction result represented by the red point in Figure 3a because the distance D1’ between one of the red points and its corresponding ground truth point is obviously greater than D2’~D6‘. From Formula (2), if the sum of D1~D6 is equal to the sum of D1’~D6’, the network trained with CD as the loss function cannot distinguish between the two reconstructions. Therefore, we can say that CD may confuse different reconstructions with similar chamfer distances. However, in the EMD loss function, *φ* represents the bijection relationship between the ground truth and the reconstructed point cloud, so EMD loss will not cause the above confusion.

EMD is related to the visual quality of the reconstructed point cloud. Lower EMD value always presumes higher visual quality. However, it is inclined to obtain a bad contour of the object. Synthesizing the pros and cons of CD and EMD, the loss function of our network is defined as:

(4) $$Loss=L_{CD}+L_{\mathrm{EMD}}$$

## 4. Experiment

We evaluated the proposed 3D-SSRecNet on ShapeNet [28] and Pix3D [29] datasets, respectively. ShapeNet is a big collection of textured CAD models which consists of 13 classes and 43,809 point cloud models for both training and testing. We used the 80–20% train/test split to perform our experiment. We performed the same experiment on the Pix3D database. The Pix3D database consists of three classes and 7595 point cloud models. This dataset is a CAD model of the real scene. Experiments on Pix3D can better evaluate the practicability of a point cloud reconstruction algorithm.

We used the gradient optimization algorithm Adam to optimize the proposed 3D-SSRecNet. In training, we set the learning rate to 0.0005 and the epoch to 50. The training environment is as follows: Ubuntu 18.04.6, CUDA 10.1, and the model of GPU is NVIDIA Tesla T4 × 4. We used the CD and EMD values calculated on 1024 sampling points to evaluate the quality of the reconstructed point cloud.

### 4.1. Experiment on ShapeNet

To verify the advantage of ELU activation function, we fixed the structure of 3D-SSRecNet and replaced ELU with other activation functions such as Leaky ReLU, softsign, and softplus. Table 2 shows the reconstruction results using different activation functions. Among all the 13 classes, ELU performed better in 12 categories.

**Figure 4:**
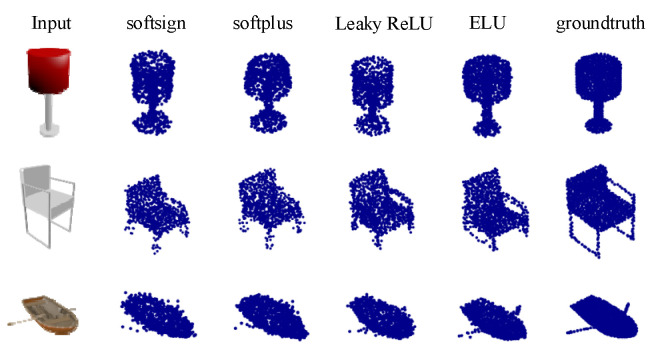
Visualization of 3D-SSRecNet’s output with different activation functions on ShapeNet.

Figure 4 shows the point cloud reconstruction results obtained using different activation functions on the ShapeNet dataset. We can see that the network trained by ELU considers details better and preserves finer geometric information.

We compared our 3D-SSRecNet with PSGN [4], 3D-LMNet [5], SE-MD [24], 3D-VENet [25], 3D-ARNet [22], 3D-ReConstnet [6], and 3D-FEGNet [26]. All the experiments followed the same train/test split and used the same loss functions, CD and EMD. Table 3 and Table 4 show the reconstruction results of different reconstruction methods on the ShapeNet dataset. The smaller the values of CD and EMD of a method, the better the reconstruction quality of the method. From the results shown in Table 3, the CD values of 3D-SSRecNet are slightly lower than that of 3D-Renstnet in the two categories of cabinet and monitor. In other categories, the reconstruction effect of 3D-SSRecNet is the best. In Table 3, PSGN, 3D-LMNet, SE-MD, and 3D-ARNet are two-stage networks, while other networks are single-stage networks. It can be seen from the CD values shown in Table 3 that the performance of single-stage networks in most categories is better than that of two-stage networks. This shows that the propagation of features between different stages of the network will cause feature loss. It can be seen from the EMD values shown in Table 4 that the reconstruction results of SSRecNet in most categories are better than those of other networks.

**Figure 5:**
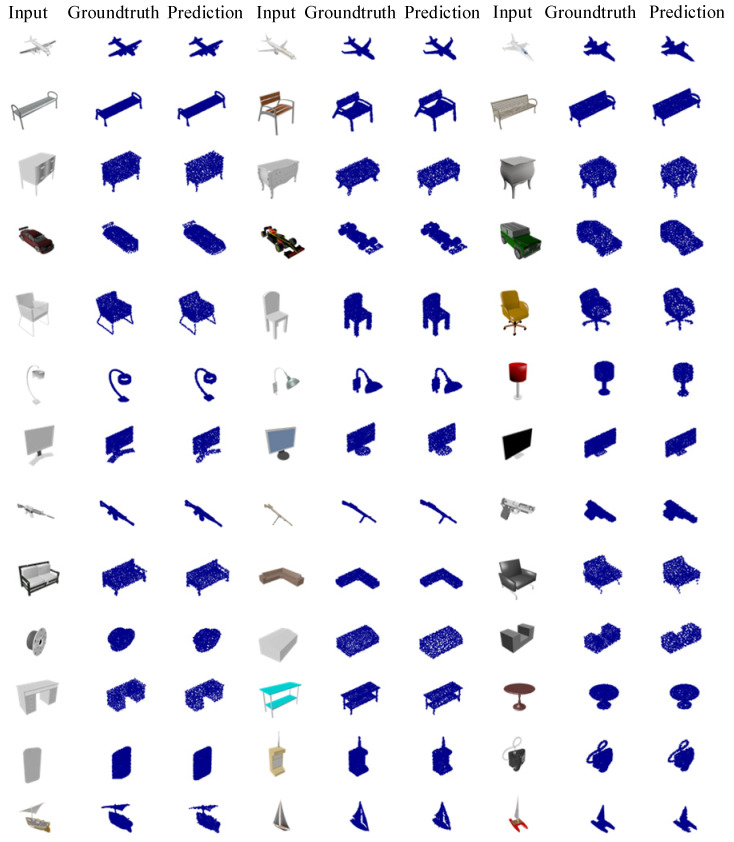
Visualization of our predictions on ShapeNet.

Figure 5 shows the reconstruction effect of 13 categories of 3D-SSRecNet on the ShapeNet dataset. The reconstructed point cloud resolution shown in Figure 5 is 2048.

### 4.2. Experiment on Pix3D

**Figure 6:**
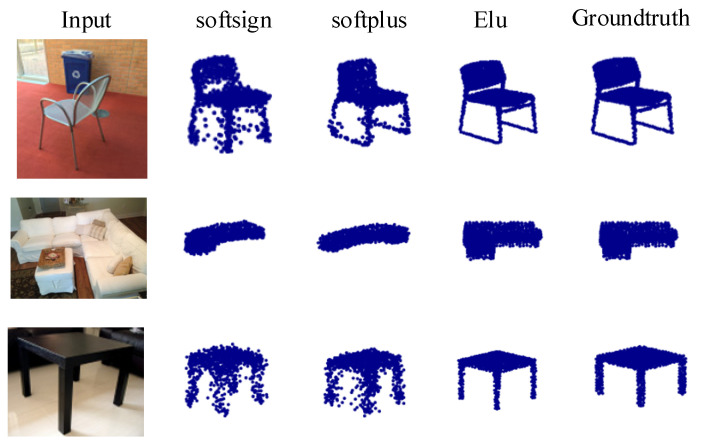
Visualization of 3D-SSRecNet’s predictions with different activation functions on Pix3D.

The Pix3D dataset consists of a large number of real indoor 2D images and their corresponding metadata (such as masks, ground truth CAD models, and attitudes). It can be seen from Figure 6 that the background of 2D images in Pix3D dataset is very complex, which poses a greater challenge to the feature extraction part of the reconstruction network. Therefore, the Pix3D dataset can be used to evaluate the generalization ability of the reconstructed network to real scenes.

Table 5 shows that ELU also provides better evaluation values on Pix3D. We exhibit the prediction outputs with different activation functions in Figure 6 to visualize how the activation function affects the prediction. Figure 6 shows that ELU also generates better qualitative results.

We also compared our network with PSGN [4], 3D-LMNet [5], 3D-ARNet [22], 3D-Reconstnet [6], and 3D-FEGNet [26]. Table 6 and Table 7 show the reconstruction results of different reconstruction methods on the Pix3D dataset. In all three categories, we can see that our 3D-SSRecNet has the lowest evaluation value (except the EMD value on chair category), which indicates that 3D-SSRecNet has a strong generalization ability for real scenes.

**Figure 7:**
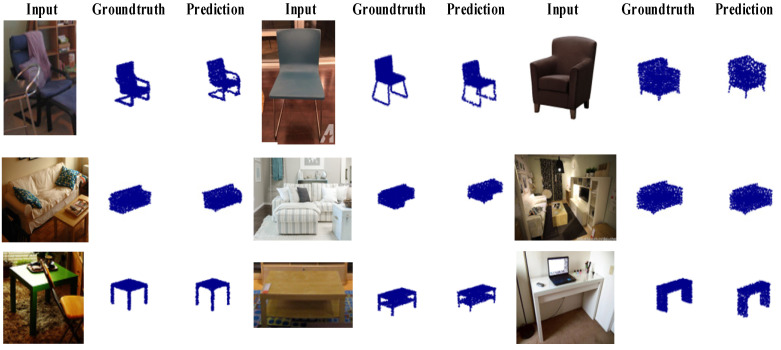
Visualization of our predictions on Pix3D.

Figure 7 shows the reconstruction effect of 13 categories of 3D-SSRecNet on the Pix3D dataset. The reconstructed point cloud resolution shown in Figure 7 is 2048.

## 5. Conclusions

In this paper, we proposed an efficient 3D point cloud reconstruction called 3D-SSRecNet. Given an image, it learns the latent representation and after dimensionality reduction we apply MLPs to predict the correspondent point cloud directly. We conducted several experiments on ShapeNet and Pix3D data sets. We proved that the reconstruction effect of using activation function ELU in the generation network is better than that of using other activation functions. That is, the CD and EMD values of the point cloud generated using the ELU are lower than those of the point cloud generated using other activation functions. Figure 4 and Figure 6 also show that the shape and contour of the point cloud generated using ELU are better than those of the point cloud generated using other activation functions. By comparing CD and EMD values, it is proved that 3D-SSRecNet can outperform the state-of-the-art methods. The proposed 3D-SSRecNet is more suitable for tasks that need to obtain the contour and shape of a point cloud at a low cost. In future work, we will try to improve the local effect of the output point cloud with the Part-Wise AtlasNet on the premise of maintaining the existing low cost.

## Figures and Tables

**Table 1:** The parameters of last two stages DetNet.

Stage	Feature Map Size	Parameters (Convolution Kernel Size, Number of Output Channels)
Stage 5	16 × 16	$\left[\begin{array}{cc} 1\times 1\mathrm{kernel} & 256chanels\\ 3\times 3\mathrm{kernel},\mathrm{dilate}\;2 & 256chanels\\ 1\times 1\mathrm{kernel} & 256chanels \end{array}\right]+1\times 1conv$ $\left[\begin{array}{cc} 1\times 1\mathrm{kernel} & 256chanels\\ 3\times 3\mathrm{kernel},\mathrm{dilate}\;2 & 256chanels\\ 1\times 1\mathrm{kernel} & 256chanels \end{array}\right]\times 2$
Stage 6	16 × 16	$\left[\begin{array}{cc} 1\times 1\mathrm{kernel} & 256chanels\\ 3\times 3\mathrm{kernel},\mathrm{dilate}\;2 & 256chanels\\ 1\times 1\mathrm{kernel} & 256chanels \end{array}\right]+1\times 1conv$ $\left[\begin{array}{cc} 1\times 1\mathrm{kernel} & 256chanels\\ 3\times 3\mathrm{kernel},\mathrm{dilate}\;2 & 256chanels\\ 1\times 1\mathrm{kernel} & 256chanels \end{array}\right]\times 2$
	1 × 1	16 × 16 average pool, 1000-d fully connected layer

**Table 2:** Reconstruction results of different activation functions on ShapeNet dataset.

Class	Softsign	Softplus	Leaky ReLU	ELU	Softsign	Softplus	Leaky ReLU	ELU
	$CD\times 10^{-2}$				$EMD\times 10^{-2}$			
Airplane	2.60	2.38	2.37	2.38	3.05	2.72	2.68	2.72
Bench	3.69	3.58	3.55	3.51	3.34	3.27	3.18	3.15
Cabinet	5.19	5.09	4.9	4.77	4.28	4.21	4.03	3.91
Car	3.58	3.57	3.57	3.56	2.85	2.85	2.85	2.84
Chair	4.45	4.48	4.40	4.35	4.30	4.36	4.23	4.17
Lamp	5.04	5.21	4.97	4.99	6.36	6.75	6.23	6.19
Monitor	5.00	4.84	4.73	4.72	4.96	4.77	4.50	4.49
Rifle	2.52	2.58	2.49	2.45	3.69	3.98	3.48	3.48
Sofa	4.57	4.62	4.52	4.44	3.82	3.89	3.73	3.71
Speaker	6.26	6.26	5.99	5.94	5.58	5.65	5.27	5.23
Table	4.86	4.83	4.40	4.35	4.75	4.74	4.21	4.16
Telephone	3.75	3.71	3.57	3.52	3.41	3.52	3.06	3.05
Vessel	3.85	3.77	3.77	3.72	4.20	4.26	3.98	3.96

**Table 3:** Reconstruction results on ShapeNet evaluated by CD.

Class	PSGN	3D-LMNet	SE-MD	3D-VENet	3D-ARNet	3D-Reconstnet	3D-FEGNet	Ours
	$CD\times 10^{-2}$							
Airplane	3.74	3.34	3.11	3.09	2.98	2.42	2.36	2.38
Bench	4.63	4.55	4.34	4.26	4.44	3.57	3.60	3.51
Cabinet	6.98	6.09	5.89	5.49	6.01	4.66	4.84	4.77
Car	5.2	4.55	4.52	4.30	4.27	3.59	3.57	3.56
Chair	6.39	6.41	6.47	5.76	5.94	4.41	4.35	4.35
Lamp	6.33	7.10	7.08	6.07	6.47	5.03	5.13	4.99
Monitor	6.15	6.40	6.36	5.76	6.08	4.61	4.67	4.72
Rifle	2.91	2.75	2.81	2.67	2.65	2.51	2.45	2.45
Sofa	6.98	5.85	5.69	5.34	5.54	4.58	4.56	4.44
Speaker	8.75	8.10	7.92	7.28	7.65	5.94	6.00	5.94
Table	6.00	6.05	5.62	5.46	5.68	4.41	4.42	4.35
Telephone	4.56	4.63	4.51	4.20	4.10	3.59	3.50	3.52
Vessel	4.38	4.37	4.24	4.22	4.15	3.81	3.75	3.72
Mean	5.62	5.4	5.27	4.92	5.07	4.09	4.09	4.05

**Table 4:** Reconstruction results on ShapeNet evaluated by EMD.

Class	PSGN	3D-LMNet	SE-MD	3D-VENet	3D-ARNet	3D-Reconstnet	3D-FEGNet	Ours
	$EMD\times 10^{-2}$							
Airplane	6.38	4.77	4.78	3.56	3.12	2.80	2.67	2.72
Bench	5.88	4.99	4.61	4.09	3.93	3.22	3.75	3.15
Cabinet	6.04	6.35	6.37	4.69	4.81	3.84	4.75	3.91
Car	4.87	4.10	4.11	3.57	3.38	2.87	3.40	2.84
Chair	9.63	8.02	6.53	6.11	5.45	4.24	4.52	4.17
Lamp	16.17	15.80	12.11	9.97	7.60	6.40	6.11	6.19
Monitor	7.59	7.13	6.74	5.63	5.58	4.38	4.88	4.49
Rifle	8.48	6.08	5.89	4.06	3.39	3.63	2.91	3.48
Sofa	7.42	5.65	5.21	4.80	4.49	3.83	4.56	3.71
Speaker	8.70	9.15	7.86	6.78	6.59	5.26	6.24	5.23
Table	8.40	7.82	6.14	6.10	5.23	4.26	4.62	4.16
Telephone	5.07	5.43	5.11	3.61	3.25	3.06	3.39	3.05
Vessel	6.18	5.68	5.25	4.59	4.05	3.99	4.09	3.96
Mean	7.75	7.00	6.21	5.20	4.68	3.98	4.30	3.93

**Table 5:** Reconstruction results of different activation functions on Pix3D dataset.

Class	Softsign	Softplus	Leaky ReLU	ELU	Softsign	Softplus	Leaky ReLU	ELU
	$CD\times 10^{-2}$				$EMD\times 10^{-2}$			
Chair	5.70	5.57	5.58	5.52	6.32	6.13	6.08	6.05
Sofa	6.22	6.26	6.09	6.04	5.12	5.24	4.92	4.90
Table	7.73	7.32	7.03	6.88	8.66	8.20	7.60	7.59

**Table 6:** Reconstruction results on Pix3D evaluated by CD.

Class	PSGN	3D-LMNet	3D-ARNet	3D-ReconstNet	3D-FEGNet	Ours
	$CD\times 10^{-2}$					
Chair	8.05	7.35	7.22	5.59	5.66	5.52
Sofa	8.45	8.18	8.13	6.14	6.23	6.04
Table	10.85	11.2	10.31	7.04	7.58	6.88
Mean	9.12	8.91	8.55	6.26	6.49	6.15

**Table 7:** Reconstruction results on Pix3D evaluated by EMD.

Class	PSGN	3D-LMNet	3D-ARNet	3D-ReconstNet	3D-FEGNet	Ours
	$EMD\times 10^{-2}$					
Chair	12.55	9.14	7.94	5.99	8.24	6.05
Sofa	9.16	7.22	6.69	5.02	6.77	4.90
Table	15.16	12.73	10.42	7.60	11.40	7.59
Mean	12.29	9.70	8.35	6.20	8.80	6.18

## Data Availability

Not applicable.
